# Economic Evaluation of Letrozole for Early Breast Cancer in a Health Resource-Limited Setting

**DOI:** 10.1155/2018/9282646

**Published:** 2018-08-02

**Authors:** Ming Ye, Jingsong Lu, Fan Yang, Bin Wu

**Affiliations:** ^1^Department of Radiation Oncology, Ren Ji Hospital, School of Medicine, Shanghai Jiaotong University, China; ^2^Department of Breast Surgery, Ren Ji Hospital, School of Medicine, Shanghai Jiaotong University, China; ^3^Department of Pharmacy, Ren Ji Hospital, South Campus, School of Medicine, Shanghai Jiaotong University, China

## Abstract

**Objective:**

Long-term aromatase inhibitor (AI) therapy is expected to improve the health outcomes with high health resource consumption in early breast cancer. The aim of the study was to assess the cost-effectiveness of letrozole for postmenopausal women with estrogen receptor positive early breast cancer in a health resource-limited setting.

**Methods:**

A Markov model was developed to project the lifetime outcomes based on the clinical course of early breast cancer. The clinical and utility data were derived from reported results. Costs were estimated from the perspective of Chinese health care. The quality-adjusted life-year (QALY) and incremental cost-effective ratio (ICER) were measured. Probabilistic sensitivity and one-way analyses were conducted.

**Results:**

Compared to 5 years of tamoxifen therapy, 5 years of AI treatment with letrozole improved the QALYs (10.44 versus 10.84) and increased the lifetime costs (CNY ¥13,613 versus CNY ¥28,797), resulting in an ICER of CNY ¥38,092 /QALY. The ICER of 5 years of letrozole versus 2–3 years of tamoxifen and then letrozole was CNY ¥68,233 /QALY. Sensitivity analyses showed that the age of initiating adjuvant endocrine therapy was the most influential parameter.

**Conclusions:**

In health resource-limited settings, adjuvant endocrine therapy with letrozole is a cost-effective strategy compared to tamoxifen in women with early breast cancer.

## 1. Introduction

Breast cancer is the most common cancer in Chinese women. Cases in China account for 12.2% of all newly diagnosed breast cancers and 9.6% of all deaths from breast cancer worldwide [1]. According to the Global Burden of Disease (GBD) 2013, the disability-adjusted life years (DALY) of Chinese breast cancer increased from 1062.6 thousand in 1990 to 1,666.0 thousand in 2013, and the peak age-specific DALY rate was at 50 to 59 years [2]. Because of the lack of funding and concern about false-positive diagnoses of annual mammography, no national screening program for breast cancer was available in China. A nationwide survey in China found that 15.7% of patients were diagnosed at stage I, 44.9% at stage II, 18.7% at stage III, 2.4% at stage IV disease, and 18% at unknown stage [3]. For women with early-stage breast cancer, the primary treatment goals are recurrence control and improvement in the quality of life [4].

Endocrine therapy is one of the therapeutic backbones for early breast cancer with hormone-receptor positivity. One recent meta-analysis with individual data on 31,920 postmenopausal women with estrogen receptor positive (ER+) early breast cancer found the 10-year recurrence risks for 5 years of AIs and 2–3 years of tamoxifen and then AIs were 3.6% (95% confidence interval [CI]: 1.7–5.4%, p<0.00001) and 2.0% (95% CI: 0.2–3.8%, p=0.0001) lower than 5 years of tamoxifen, respectively [5]. Clinical guidelines have recommended the use of tamoxifen and aromatase inhibitors (AI) as adjuvant endocrine therapy options for postmenopausal women with ER(+) breast cancer [6, 7]. However, the economic status was one of the key factors affecting the use of AIs as endocrine therapy. Based on a Chinese nationwide, multicenter, 10-year retrospective clinical study, nearly 80.3% of women receiving endocrine therapy were given anti-estrogen agents and 15.5% were given AIs [8]. Several published studies compared the cost-effectiveness of adjuvant endocrine therapy for early breast cancer [9], which indicated that AIs are a cost-effective alternative. However, most of these studies came from high-income countries. In recent years, AI has been widely prescribed in Chinese clinical practice, including generic letrozole whose price is approximately 30% of the branded drug price.

The aim of this analysis was to test the cost-effectiveness of adjuvant endocrine therapy with AI for postmenopausal women with early ER (+) breast cancer compared to the current standard of tamoxifen therapy in China, a typical health resource-limited setting.

## 2. Methods

### 2.1. Economic Model Overview

**Figure 1:**
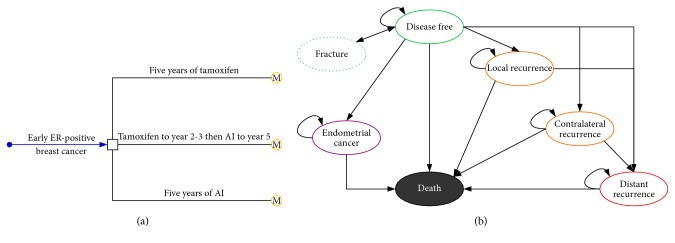
The diagram of model structure. Decision tree (a) and Markov model (b).

A mathematical model was established to measure the clinical and economic outcomes of adjuvant endocrine therapy for postmenopausal women with early ER (+) breast cancer after lumpectomy. Patients were considered to either start standard adjuvant endocrine therapy based on five years of tamoxifen (tamoxifen strategy) or to start one of the following strategies, as shown in Figure 1(a): five years of AI (AI 5-year strategy) and tamoxifen to years 2-3 and then AI to year 5 (AI switch strategy). The current analysis used letrozole as representative of AI because it is the most widely prescribed agent in Chinese practice. Because this adjuvant endocrine therapy has been recommended as the standard treatment for newly diagnosed patients with early ER (+) breast cancer by clinical guidelines [10, 11], and the aim was to evaluate the economic outcome of a different adjuvant endocrine regimen, a “no treatment” strategy was not evaluated in this study. Health and economic outcomes were predicted using the Markov state transition model (Figure 1(b)) with the following six exclusive Markov health states: being disease-free, local recurrence, contralateral recurrence, distant recurrence, endometrial cancer, and death. Bone fracture was used as a temporary health state. A hypothetical postmenopausal women cohort with confirmed newly diagnosed early ER (+) breast cancer was created for comparing three AIs with a control strategy. We set the characteristics of the hypothetical cohort to be similar to our previous study [12], which showed the age of postmenopausal women with early ER (+) breast cancer was 57.3 years (range: 27–79 years). A lifetime timeframe (i.e., until death or 100 years) was used in the model because the long-term survival of patients with early breast cancer is considerable [13]. The Markov cycle length was one month. In each cycle, the model redistributes the hypothetical women among the six health states according to transition probabilities. The initial state is assumed to be disease-free. Death is the terminal state, and the woman could die from breast cancer or other causes. The risk of cancer recurrence or death was determined by the reported literature [5, 14]. This economic analysis was based on a literature review and an experimental model, and it did not require approval from the Institutional Review Board/Ethics Committee.

The following outcomes were examined: progression-free LYs, overall LYs, QALYs, and cost. Cost and QALYs were discounted 5% annually as our previous evaluation [15]. The costs are shown as Chinese currency (RMB, ¥). ICERs, presented as the cost per additional QALY gained, were also examined. When the ICER was lower than the 3×the per capita gross domestic product (GDP) of China per QALY (¥171,000), cost-effectiveness was considered [16].

### 2.2. Clinical Data

Table 1 summarizes the model input clinical parameters and corresponding data sources, which were derived from published randomized clinical trials or meta-analyses, whenever possible. The long-term risks of local, contralateral, and distant recurrence for the tamoxifen, AI 5-year and AI switch strategy were derived from the previously described meta-analysis that was recently reported by EBCTCG [5]. This report was used as the only source of our baseline estimates of adjuvant endocrine therapy efficacy because it supplied the strongest evidence [17]. To extrapolate the risk of recurrence beyond the observational period, local, contralateral, and distant recurrence data were fitted using a parametric survival model. An exponential model was selected for fitting local and distant recurrence, and a two-parameter Weibull survival model was selected for contralateral recurrence based on the test results of goodness of fit [18]. The estimated parameters for the exponential and Weibull model are shown in Table 1. Although the meta-analysis reported the RRs of recurrence as AI 5-year strategy versus tamoxifen, the AI switch strategy versus tamoxifen, and AI 5-year versus AI switch strategy, it did not report the results of simultaneously pooling the data of three strategies. A network meta-analysis was used for recalibrating the RRs of recurrence of AI 5-year strategy versus tamoxifen and AI switch strategy versus tamoxifen by simultaneously pooling the reported RRs of three strategies [19]. We assumed that the decreased recurrence risks resulting from AI 5-year and AI switch strategy versus tamoxifen strategy were maintained over the subject's lifetime.

The risk of distant recurrence in women with local and contralateral recurrence was derived from the Chinese study reported by Wang HT. et al. [20] The Weibull survival model based on the survival curves was used for estimating the transition probabilities. We assumed the risk was equal regardless of the initial therapy. In patients with distant recurrence, the monthly probability of dying from breast cancer was nearly 2.88% based on our previous study [21].

The transition probabilities of fractures and endometrial cancer in tamoxifen strategy were also obtained from the reported results by EBCTCG [5], and they were adjusted for the AI 5-year and AI switch strategies based on their RRs. When endometrial cancer after breast cancer in relation to endocrine therapy occurred, the survival rate was estimated from a literature by pooling results from three countries [22]. The monthly probability of dying from endometrial cancer was approximately 0.98%. Monthly age- and female-specific mortality rates from other causes were based on the 2009 Chinese life tables reported by the World Health Organization (WHO) [23].

### 2.3. Cost and Utility Data

The analysis involved a heath care perspective, including direct medical costs to the health care system and the patient, such as adjuvant endocrine therapy, follow-up, adverse events, and management of recurrence of breast cancer (Table 1). Indirect cost, such as productivity costs to patients or caregivers, was not considered. The doses of tamoxifen and letrozole for adjuvant endocrine therapy were 20 mg and 2.5 mg per day, respectively. Because their generics have been widely used in Chinese setting, the daily costs were estimated based on the local prices of the generics [24]. The costs of local, contralateral, and distant recurrences and fractures and endometrial cancer were taken from published studies from China [25]. The costs of managing fractures related to osteoporosis were taken from a Chinese disease burden of osteoporosis [26]. The cost of endometrial cancer was derived from an epidemiological survey based on the 493 Chinese patients with endometrial cancer [27]. Because of the bias of local utilization reflecting national patterns, the cost impact was examined in a one-way sensitivity analysis. All costs were expressed as 2016 Chinese Yuan (CNY ¥).

The utility scores associated with each health state (Table 1) were obtained from the literature and varied from 1.0 (perfect health) to 0.0 (death) [28–31]. When a woman incurred a fracture in the disease-free state, the utility for joint health states was calculated based on the following formula: U(ij) = U(min) - U(min) (1 - U(i))(1 - U(j)) [32].

### 2.4. Sensitivity Analyses

To address the uncertainty of the model, probabilistic sensitivity analysis (PSA) was conducted using a second-order Monte Carlo technique. In this analysis, statistical distributions were adopted to the corresponding model parameters and values sampled by 1,000 Monte Carlo simulations for jointly examining the uncertainty in all model parameters. A beta-distribution was used to represent the uncertainty in utility, probability, and proportions because these are binomial parameters that are constricted in the interval from zero to one. A lognormal distribution was used for cost data. Based on the results of PSA, a cost-effectiveness acceptability curve was plotted to show the proportion of cost-effective simulations at different levels of willingness to pay per QALY gained. Moreover, one-way sensitivity analyses were used to examine the robustness of the results by changing individual variables between the lower and upper limits as shown in Table 1. All statistical calculations were performed using the R software package (version 3.3.3; R Development Core Team, Vienna, Austria).

## 3. Results

### 3.1. Base-Case Analysis

The outcomes of the three strategies in the base-case analysis are shown in Table 2. In the tamoxifen strategy group, 57.63% and 1.97% of patients developed breast cancer and endometrial cancer recurrence in their lifetime, respectively; the calculated total cost, QALYs, and expected life years per person were CNY ¥ 13,613 10.44 and 18.34, respectively. Compared with tamoxifen and the AI switch strategy, the AI 5-year strategy had a lower incidence of recurrence of breast cancer and endometrial cancer, more health benefits, and higher costs, which resulted in the ICERs of CNY ¥ 38,092 and 68,233/QALY, respectively.

### 3.2. Sensitivity Analysis

**Figure 2:**
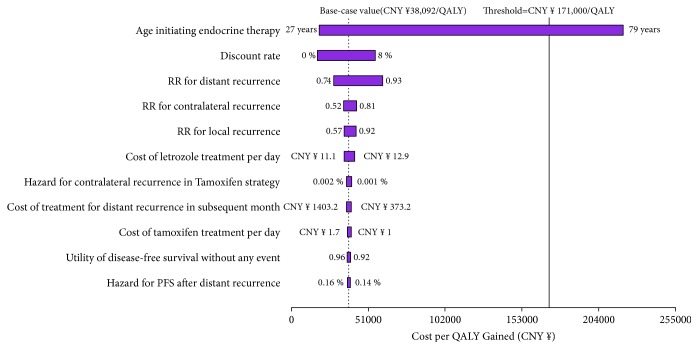
Tornado diagram representing the cost per QALY gained in one-way sensitivity analysis for AI 5-year strategy versus tamoxifen strategy. The width of the bars represents the range of the results when the variables were changed. The vertical gray and black dotted lines represent the base-case results and threshold, respectively. The width of the bars represents the range of results when the variables are changed. The vertical dotted line represents the base-case results.

The one-way sensitivity analyses revealed that the results of the model were more sensitive to the patient age because this variable had the greatest impact on ICER, which showed that the AI 5-year strategy would become more unfavorable as the patient aged. The remaining sensitive variables, such as the discount rate and RR for distant recurrence, had a medium effect (Figure 2). It is worth noting that, in all adjustments for the remainder of the variables, the AI 5-year strategy maintained its cost-effectiveness and the ICER per QALY gained remained well below the threshold of CNY ¥171,000.

**Figure 3:**
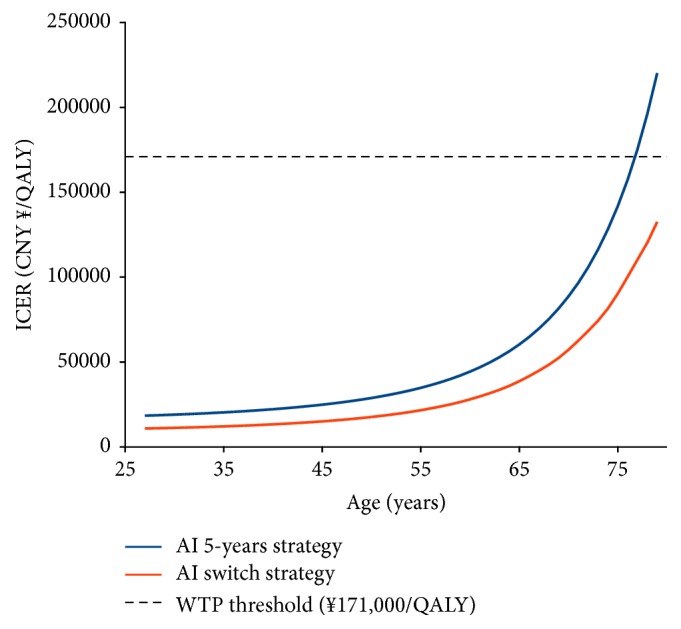
Impact of age on the ICERs of AI 5-year strategy and AI switch strategy versus tamoxifen strategy.

Across the age from 27 to 19 years old, the ICERs of AI 5-year strategy over tamoxifen strategy would exceed the threshold of CNY ¥171,000 once the age >75 years old (Figure 3). However, AI switch strategy would keep to be cost-effective.

**Figure 4:**
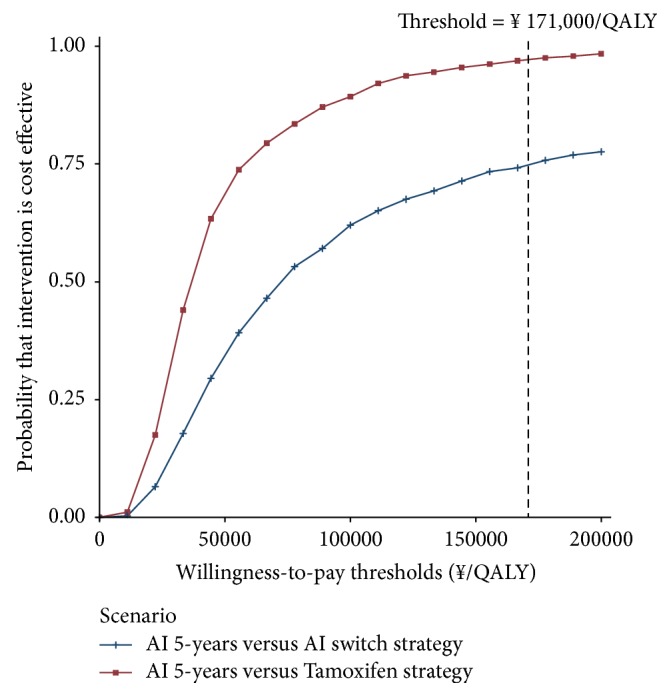
Cost-effectiveness acceptability curves of AI 5-year strategy and AI switch strategy in comparison with tamoxifen strategy. The y-axis indicates the probability that a strategy is cost-effective across the willingness to pay per QALY gained (x-axis). The vertical dashed line represents the thresholds for China.

Compared to the tamoxifen strategy and AI switch strategy, the cost-effectiveness acceptability curves showed that the AI 5-year strategy produced nearly 90% and 70% probabilities of cost-effectiveness when the threshold was equal to the 3×per capita GDP of China in 2016, respectively (Figure 4).

## 4. Discussion

To the best of our knowledge, this is the first economic evaluation of adjuvant endocrine therapy with generics for postmenopausal women with estrogen receptor positive early breast cancer in a health resource-limited setting. Our study indicates that adjuvant endocrine therapy with the AI 5-year strategy offers greater health benefits and higher cost compared to the tamoxifen and AI switch strategy. In terms of health outcomes, the strategies with AI were more effective because they reduced the frequency of disease recurrence and endometrial cancer compared with the tamoxifen strategy, resulting in higher QALYs as well as offsetting the higher cost of AI by reducing the health resource utilization of the complications (Table 2). Hence, the AI 5-year strategy was found to be a cost-effective alternative, resulting in an ICER equal to CNY ¥ 38,092 and 68,233 per additional QALY gained compared to the tamoxifen and AI switch strategies, which were lower than ¥ 57,000 (per capita GDP of China in 2016) and ¥ 171,000 (3×per capita GDP of China in 2016), respectively. Based on the standard recommended by the WHO CHOosing Interventions that are Cost-Effective (WHO-CHOICE) project [33], these results indicate that the AI 5-year strategy with letrozole might be a very cost-effective option compared to the tamoxifen strategy as well as a cost-effective option compared to the AI switch strategy in a health resource-limited setting.

Several studies have investigated the economic outcomes of using adjuvant endocrine therapy among women with estrogen receptor positive early breast cancer [9]. These reports showed that the ICERs for the five years of AIs versus five years of tamoxifen ranged from $24,109 to $61,278 per life-year and from $25,886 to $59,620 per QALY gained. All reported ICERs were less than $100,000 per life years and the majority were less than $50,000 per QALY, indicating that adopting aromatase inhibitors as a first-line therapy was cost-effective compared to tamoxifen from the perspective of North America and Europe. Our results from the current analysis agreed with these publications. However, our ICER was lower than the reported results. One of the main reasons is that the price of letrozole was lower than the branded agents. The median price difference of letrozole versus tamoxifen was CNY ¥ 10.5(~$1.50) per day, and the difference of the branded agents versus tamoxifen was ~$3.50–5.50 per day. When branded AI agents were used, two studies from the United States and Colombia found tamoxifen to be the cost-effective option for adjuvant therapy lasting five years [34, 35]. In China, the branded AI agent is expensive. For example, branded letrozole is about CNY ¥47.3 per day, which would lead the ICERs of AI 5-year strategy and AI switch strategy over tamoxifen strategy to be CNY ¥ 176,885 and CNY ¥ 115,865/ QAYL, respectively. These results indicated that AI 5-year strategy is no longer to be cost-effective.

One-way sensitivity analysis found that the age of initiating adjuvant endocrine therapy was the most influential parameter. This result indicates that the younger subgroup can gain more monetary value from the adjuvant endocrine therapy with letrozole. One economic study from a Canadian perspective reported by Delea TE et al. also found that adjuvant endocrine therapy with letrozole may be more cost-effective in younger patients [36]. Other independent and influential parameters include the risk ratio of distant recurrence. A lower risk ratio of distant recurrence will have favorable economic outcomes for adjuvant endocrine therapy with letrozole. Women with small tumor size and low lymph node category might gain more monetary value from letrozole therapy due to their lower risk ratio of distant recurrence [37].

The present analysis has several limitations. First, parametric survival models were used for extrapolating the clinical benefit beyond trial observation, which is an inevitable limitation in this analysis. There might be much uncertainty in the lifetime survival probability, although the present model did not show that the PFS and OS had considerable impacts on the outcome. Second, due to the lack of well-designed clinical trials in the Chinese population, the recurrence data were derived from abroad for the model key inputs. However, one study from East Asia suggested that there is no notable difference in the efficacy of adjuvant endocrine therapy for early breast cancer data among different regions [38]. The results from sensitivity analyses found that the model outcomes were robust. Third, the tremendous advances over the last two decades, such as targeted therapy in surgery, have significantly increased breast cancer survival [4]. The risk of recurrence with adjuvant endocrine therapy may be notably different in future practice. To simplify our analysis, we did not account for this issue. Fourth, the age-specific rates of all-cause mortality in women with breast cancer were considered similar to those in the Chinese general population in the present study. Adjuvant endocrine therapy may increase the risk of mortality from heart disease and secondary neoplasms, although only a slight impact was observed (0.2% excess mortality) [13]. Fifth, we did not perform a budget impact analysis of the adjuvant endocrine therapy with adjuvant endocrine therapy. The age-standardized mortality was 21.6 per 100,000 females [1], and letrozole might be prescribed to more than 50,000 patients annually. Based on the results from our model, the AI 5-year strategy with letrozole will increase expenditures by approximately CNY ¥ 180 million. Finally, for simplicity, the long-term impact of fractures on the costs and utilities was only considered for patients in disease-free health states. The favorable outcome of AI strategies might be overestimated. Based on the results of sensitivity analysis, this simplification will not materially affect our findings. Because of these shortcomings, the results should be carefully explained when they are referenced by local decision makers.

To conclude, our finding indicates that the AI 5-year strategy with letrozole is cost-effective compared to tamoxifen and the AI switch strategy among postmenopausal women with early breast cancer from the Chinese healthcare system perspective, which should be considered for coverage in health resource-limited settings.

## Figures and Tables

**Table 1:** Model inputs.

Parameters	Base-case Value	Range Tested	Reference
Clinical data			
Exponential distribution of distant recurrence in Tamoxifen strategy	*λ*=0.001524	0.001423 - 0.001625	[5]
Exponential distribution of local recurrence in Tamoxifen strategy	*λ*=0.000292	0.00029 - 0.000294	[5]
Weibull distribution of contralateral recurrence in Tamoxifen strategy	*λ*=0.000017; *γ*=1.6165	*λ*=0.000013 - 0.000021	[5]
RR for recurrence (AI 5-years versus Tamoxifen strategy)			
Distant recurrence	0.830	0.74 - 0.93	[5]*∗*
Local recurrence	0.7200	0.57 - 0.92	[5] *∗*
Contralateral recurrence	0.6500	0.52 - 0.81	[5] *∗*
RR for recurrence (AI switch versus Tamoxifen strategy)			
Distant recurrence	0.90	0.8 - 1.01	[5]*∗*
Local recurrence	0.84	0.68 - 1.03	[5] *∗*
Contralateral recurrence	0.63	0.51 - 0.8	[5] *∗*
Probability of death for distant recurrence	0.0290	0.023 - 0.041	[21]
Weibull distribution of PFS after local recurrence	*λ*=0.0458; *γ*=0.782	*λ*=0.004498 - 0.023713	[20]
Weibull distribution of PFS after contralateral recurrence	*λ*=0.0487; *γ*=0.742	*λ*=0.044114 - 0.053286	[20]
Probability of endometrial cancer in Tamoxifen strategy	0.00010	0.000084 - 0.000117	[5]
Probability of fracture in Tamoxifen strategy	0.00047	0.000375 - 0.000551	[5]
RR for endometrial cancer in AI treatment	0.33	0.21 - 0.51	[5]
RR for fracture cancer in AI treatment	1.42	1.28 - 1.57	[5]
Probability of death for endometrial cancer	0.0098	0.0091 - 0.0105	[22]
Preference weights (Utility)			
DFS without any event	0.94	0.92 - 0.97	[28, 29]
Endocrine therapy	0.01	0.01 - 0.01	[28, 29]
Fracture	0.70	0.64 - 0.96	[30]
Regional recurrence	0.78	0.77 - 0.79	[28, 29]
Distant recurrence	0.53	0.42 - 0.64	[28, 29]
Endometrial cancer	0.83	0.68 - 0.95	[31]
Resource utilization and cost data (CNY ¥)			
Tamoxifen generic treatment per day	1.4	0.98 - 1.67	Local charge
Generic letrozole treatment per day	11.9	11.08 - 12.9	Local charge
Treatment for local and contralateral recurrence in the first month	75421	44364 - 106478	
Treatment for local and contralateral recurrence in subsequent months	865	509 - 1221	[25]
Treatment for distant recurrence in the first month	83008	34876 - 131140	[25]
Treatment for distant recurrence in subsequent months	888	373 - 1403	[25]
Treatment for endometrial cancer per patient	17138	14499 - 19776	[27]
Treatment for fracture per event	25552	21440 - 30622	[26]

*∗* The RRs were recalibrated based on the reported data [5] using network meta-analysis.

AI: aromatase inhibitor; RR: risk ratio.

**Table 2:** Clinical and economic outcomes of base-case patients.

Strategies	Tamoxifen strategy	AI 5-year strategy	AI switch strategy
Cost (CNY ¥)	13,613	28,797	20,061
QALYs	10.44	10.84	10.71
Life years	18.34	19.17	18.91
Cumulative probabilities of clinical events			
Recurrence of breast cancer	57.63%	47.25%	49.36%
Endometrial cancer	1.97%	0.70%	0.88%
ICER (CNY ¥/QALY, AI 5-year versus Tamoxifen strategy)	NA	38,092	NA
ICER (CNY ¥/QALY, AI 5-year versus AI switch strategy)	NA	68,233	NA

AI: aromatase inhibitor; NA: not applicable.

## Data Availability

No data were used to support this study.
